# Effectiveness of PUSH notifications from a mobile app for improving the body composition of overweight or obese women: a protocol of a three-armed randomized controlled trial

**DOI:** 10.1186/s12911-020-1058-7

**Published:** 2020-02-24

**Authors:** A. Hernández-Reyes, G. Molina-Recio, R. Molina-Luque, M. Romero-Saldaña, F. Cámara-Martos, R. Moreno-Rojas

**Affiliations:** 1 Department of Bromatology and Food Technology, University of Córdoba, Campus Rabanales, ed. Darwin - annex. Office of Dr. Rafael Moreno, 14071 Córdoba, Spain; 2 Nursing department, University of Medicine and Nursing of Córdoba, Córdoba, Spain; 3 Department of Occupational Health and Safety, Córdoba, Spain

**Keywords:** mHealth, Obesity, Weight management, Physical activity

## Abstract

**Background:**

The penetration level of mobile technology has grown exponentially and is part of our lifestyle, at all levels. The use of the smartphone has opened up a new horizon of possibilities in the treatment of health, not in vain, around 40% of existing applications are linked to the mHealth segment. Taking advantage of this circumstance to study new approaches in the treatment of obesity and prescription of physical activity is growing interest in the field of health. The primary outcome (obese adult women) will be assessed according to age, fitness status, weight, and body composition status. Data will be collected at enrollment and weekly during 6 months of intervention on dietary practices, physical activity, anthropometry, and body composition. Analysis of effect will be performed comparing the outcomes between intervention and control arms. The message delivery is in progress.

**Methods:**

A 3-arm clinical trial was established. A series of quantitative and qualitative measures were used to evaluate the effects of self-weighing and the establishment of objectives to be reached concerning the prescription of physical activity. At the end of this pilot study, a set of appropriate measures and procedures were identified and agreed upon to determine the effectiveness of messaging in the form of PUSH technology. The results were recorded and analyzed to begin a randomized controlled trial to evaluate the effectiveness of the proposed methodology.

**Conclusions:**

The study is anticipated to establish feasibility of using PUSH notifications to evaluate whether or not an intervention of 6 months, directed by a team formed by Dietician-Nutritionist and nursing professionals, by means of an application for Smartphone and a personal consultation, improves the body composition of adult women with a fat percentage equal to or higher than 30% at the beginning of the study.

**Trial registration:**

Clinical Trials ID: NCT03911583. First Submitted: April 9, 2019. Ethical oversight is provided by the Bioethical Committee of Córdoba University and registered in the platform clinicaltrials.gov. The results will be published in peer-reviewed journals and analysis data will be made public.

## Background

The use of mobile technology and its presence in our daily lives is increasing exponentially. It has been estimated that, in 2019, worldwide, there will be more than 2700 million Smartphone users, and around 1400 million Tablet owners [1]. Also, the technical improvements in mobile devices, including larger displays and a higher resolution, an increase in surfing speeds and the development of an infinity of applications (APP) with a multitude of new functions [2], has signified an authentic social and cultural revolution, reaching all society levels. As a result, the incorporation of mobile technology into our daily habits has triggered changes in the way that we live, in our work, or in how we communicate and relate to each other socially [3].

According to the Global System Mobile Association (GSMA), there are more devices connected to the network than people in the world. In 2017, 7422 million mobile connections were identified, whereas the world population census was of 7228 million [4]. Another relevant fact that helps to size up the magnitude of this technological tendency is that, in 2014, the number of accesses and browsing time in the web through mobile devices exceeded, for the first time, those made by office desk equipment [3, 5–7]. The future of technology and the mobile phone are considered to be on equal terms, making it very difficult to distinguish between one and the other. Thus, it is believed that, in a few years, we shall be able to dispense with the adjective “mobile” when speaking about technologies as they will all have this characteristic [3].

The term mHealth (mobile health) was used and defined for the first time in 2000 [8]. This concept was subsequently employed in the 2010 mHealth Summit of the Foundation for National Institutes of Health (FNIH) to refer to “*the provision of medical attention services through mobile communication devices*” [9] and nowadays this is globally understood as a medical and public health practice based on the use of mobile devices [10]. Since then, up to now, around 40% of the over 300,000 applications available in the different apps stores are related to health themes, with those focused on the monitoring and management of diseases standing out [11]. Different strategies, going from phone calls or sending information through the Short Message Service (SMS), up to the use of applications such as those for clinical decision-making support or telemedicine, have shown themselves to be effective in the communication between patients and health professionals; the change toward healthy lifestyles (giving up smoking or increasing physical exercise); in the improvement of illness management (in diabetes or asthma, for instance); and in the increase in adherence to the treatments [12–15].

One of the characteristics of mobile applications is the sending and receiving of messages through a system of notifications known as “PUSH,” that consists of requests appearing on the display of the Smartphone at a scheduled time, permitting them to be customizable both in their contents and at the time of sending them. Their principal difference from SMS lies in the fact that the latter are asynchronous, i.e., it is not expected or required for the recipient to answer a message. However, PUSH notifications are pro-active as they offer visual or aural alerts to inform the recipient of a message or event received and invite them to act on them [16]. On receiving the notification, the user can interact in different degrees, from merely reading it to answering it, thus permitting feedback. Also, there is evidence of the PUSH notifications being effective in communications between professionals [17].

SMSs have demonstrated that they are an excellent resource for delivering electronic reminders in practice and a very feasible platform, being useful in increasing adherence to treatment [18], preventing complications in non-communicable diseases [19], facilitating inter-professional communication [20], and helping in disease self-management [21]. PUSH notifications (defined as an event-based mechanism where remote servers “push”/CONVEY events to Smartphone client apps) [22] has recently appeared in mhealth, showing its potential for improving pervasive functionalities in mobile health apps, allowing the delivery of timely updates and customized reminders to users or patients. One of the essential functionalities is to offer alerts to inform the user about a received message and invite him/her to act, even without needing to be the app being used [23]. Although this strategy has proved to be effective in communication with professionals [17] and assessing patterns of health behaviors [24], there is scarce evidence of its effectiveness in interventions aimed at changing lifestyles.

Interventions that use mobile and portable technologies can be useful in improving healthy habits or reducing high levels of sedentary behavior [25, 26]. It has been demonstrated that certain functions implicit in the habitual use of Smartphones, like the exchanging of information, the possibility of carrying out self-monitoring with natural, intuitive record systems, the interaction between users, or the employment of gamification strategies, also have positive effects on the health status [27].

Besides, the users should feel that they are part of the technology, it is especially important to involve patients in active commitments, like self-assessment in certain behaviours, or making timely follow-ups [28]. These measures have been seen to be efficacious in improving health markers, for example, weight management, and blood pressure [29].

The objective of a large part of the health interventions based on the use of APPs has been to improve nutritional status through dietary advice and an increase in physical activity (PA) [30]. In this sense, it has become evident that an increase in PA implies benefits to health and reduces mortality from all causes, regardless of the body mass index (BMI) [31]. Also, there is ample evidence of the role of PA in weight loss programs in the long-term prevention of recovering weight loss [32]. In past years, systematic reviews have been done in order to establish associations between physical activity and weight loss in overweight or obese individuals [33, 34], demonstrating the existence of an inverse association between the physical activity carried out and the BMI.

In this study, we aim to investigate whether mHealth that included text messages via PUSH notifications containing advice for dietary and lifestyle modifications for six months would reduce the percentage of total body fat among overweight or obese women adults aged 25–64 years in a predominantly urban Southern Spain population. The study also aims to evaluate the effects of the mHealth intervention on body mass index, dietary practices, and physical activity. As an initial hypothesis, we considered that those subjects assigned to the group receiving the PUSH notifications would adhere to the dietary recommendations and physical activity proposed, thus achieving a more significant fat loss and a higher increase in muscle mass.

## Methods/design

### Study design

A controlled randomized three-armed clinical trial was conducted that includes a physical activity lasting six months in women following the same dietary prescription. The groups were differentiated based on whether or not they received Push notifications from a mobile application (Nutrición Sur version 15.0.0). Thus, the control group did not receive these notifications, whereas the women who received them constitute an experimental group. Additionally, inside each group, three different subgroups were randomly established with distinct degrees of physical activity (PA) intensity; these could be light (LPA), moderate (MPA), or intense (IPA).

### Sample size calculation

The primary outcome variable was fat mass loss after six months, and the anticipated minimum difference in the average fat mass loss was 2%, with an expected SD not exceeding 3.5% [35]. The study was designed to have at least an 80% power and an alpha level set at 0.5, obtaining a sample size of 27 individuals for each group (total *N* = 54). A total of 90 women (45 for each group), was estimated, to mitigate the effect of possible dropouts during this trial.

### Eligibility criteria (inclusion and exclusion)

The women with the following pathologies or special situations was excluded from the study: Type 2 Diabetes, being or trying to become pregnant, being in a maternal lactation period, suffering from kidney failure, being under age, presenting a healthy weight (BMI ≤ 25) or receiving pharmacological antidepressant treatment. Women not possessing smartphones with Android or iOS operating systems and those without available data connections did not participate in the study.

**Fig. 1:**
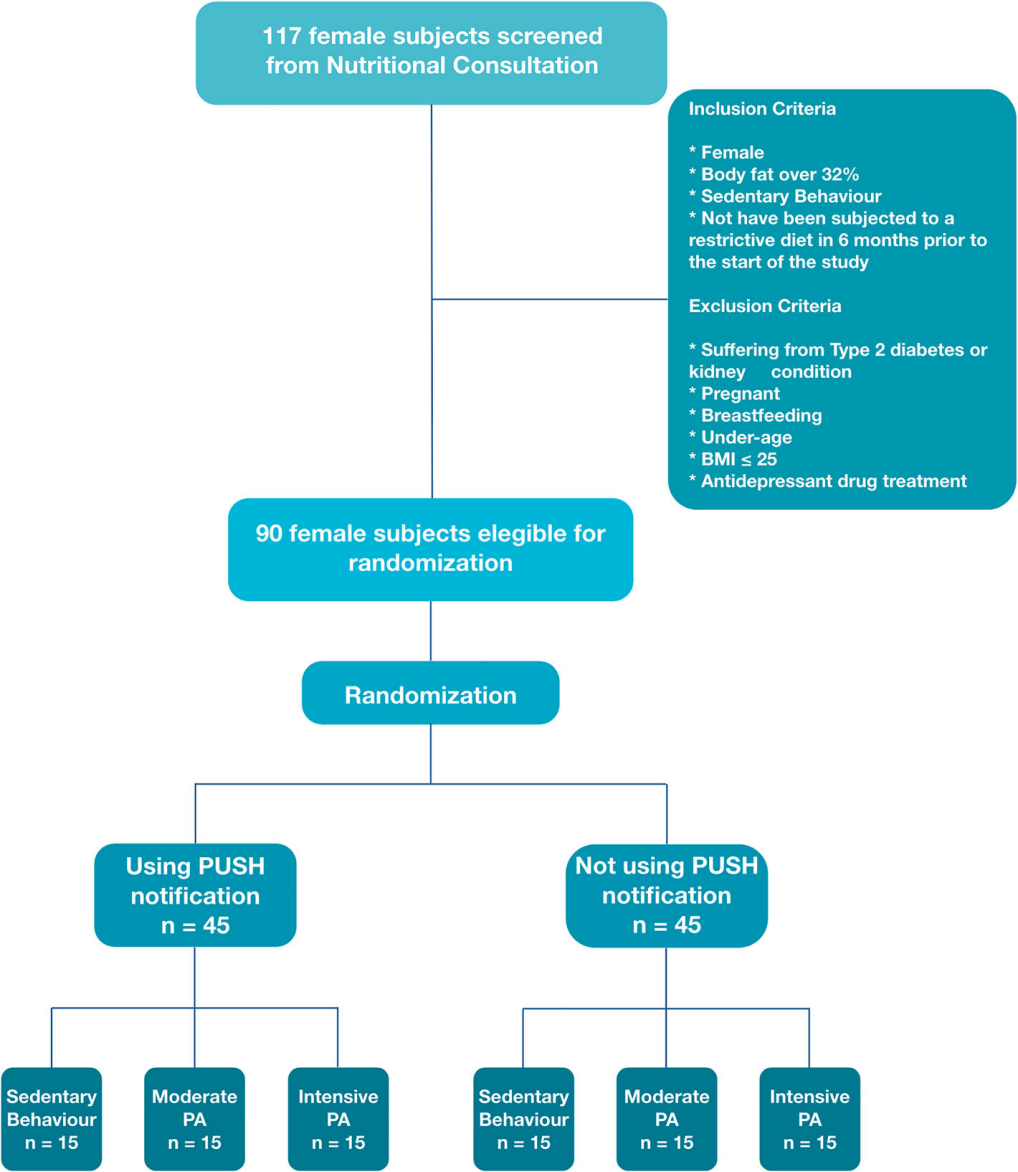
Flow chart of the participants

Also, with the aim of homogenizing the study population, the inclusion criteria were: having a body fat percentage of ≥30%, being sedentary, defined as low energy sitting (or reclining) during waking hours [36], and not having been submitted to a restrictive diet in the 6 months prior to the beginning of the study. The flow chart of the participants is found in Fig. 1.

### Study variables and measurements

#### PUSH notifications

**Fig. 2:**
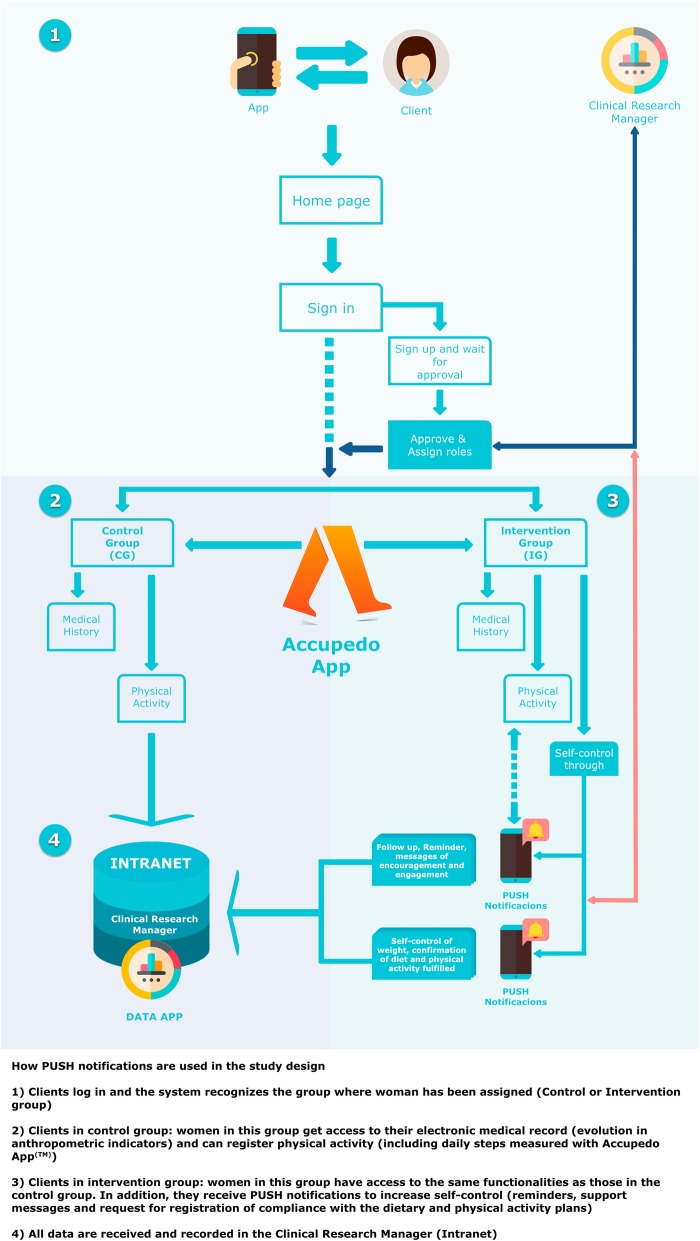
Implementation of the PUSH notifications in the study design

Figure 2 shows the implementation of the PUSH notifications in the study design. Automatic PUSH notifications were scheduled to be sent on specific days, or not, with personalized health and motivation messages, which was aimed to provide comments for reinforcing behavior modification and encouraging interaction with the APP. These comments were based on the following behavioral theories:

- Health tips, where the primary tailoring goals were: attention and peripheral processing [37].
- Physical Activity Tips, in this case: attention and being informed [38].
- Self-monitoring tips, where the primary tailoring goals were: decision making and behavioral intention [39].

Three specific times were established throughout the day for the sending of messages. According to previous works [38, 39], the best time for sending PUSH notifications was depended on: a) when patients were able to fix their preferred time for receiving them, b) trying to deliver them at times that did not interrupt the daily routine (notifications were more effective) [40]. For these reasons, it was determined that the best adherence was achieved at the times of day at which there were no commitments (before work, during lunch, before supper), so we fixed for 8.30 a.m. (point 1), 14.00 p.m. (point 2) and 20.00 p.m. (point 3). The first message was sent between points 1 and 2, and those users who do not answer it again received an automated notification in point 3.

**Fig. 3:**
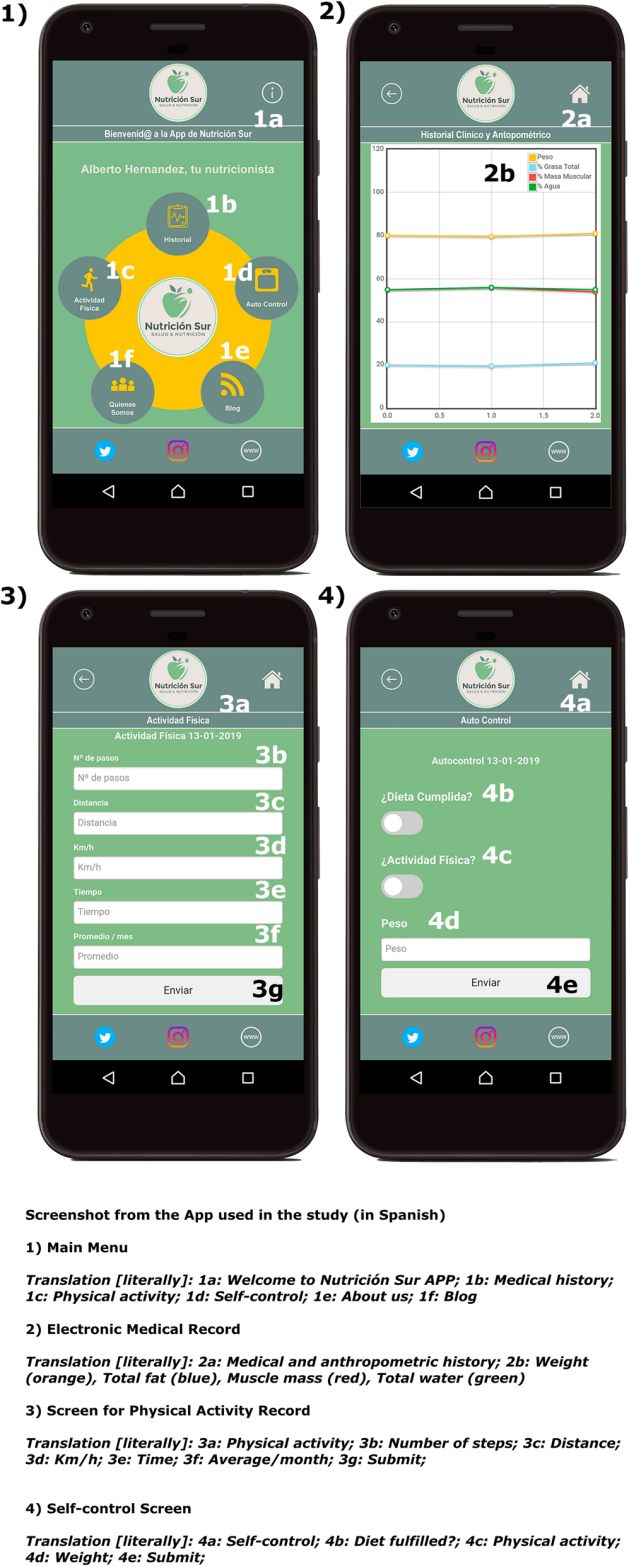
Screenshot of the APP developed for the research project (Nutrición Sur version 15.0.0)

**Fig. 4:**
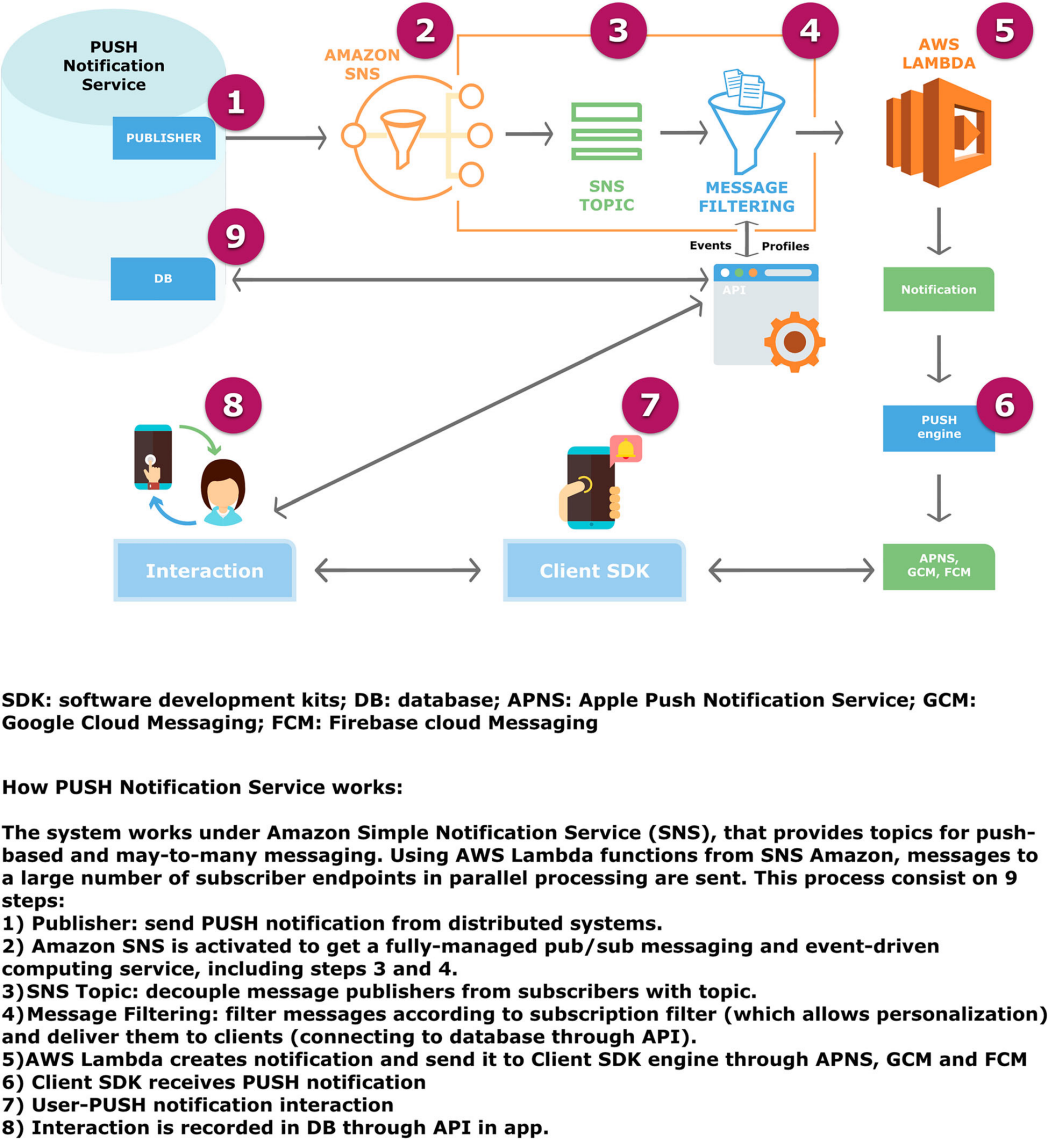
PUSH notifications architecture

The App Nutrición Sur (Fig. 3) sent automatic notifications (see architecture in Fig. 4) programmed to receive on concrete days, with personalized messages on health and motivation. The contents of the messages were extracted from a previously established library that is consistent with advice related to food consumption and physical activity. This section aimed to stimulate and remind the patient about the protocol assigned, encourage her to complete a specific steps objective (that she should be reported in the App), or carry out a session at her sports center. Also, the App included a Self-monitoring menu in which the patient could give her opinion on the diet proposed, on doing the physical activity prescribed and on her body weight, measured on home scales. The objective was to determine the effect of the PUSH notifications on compliance with the intervention protocols, as well as the changes in body composition. The information supplied in the two previous measures appeared in real-time on the researcher’s internet control panel.

#### Body composition

The percentage of body fat (BF), muscle mass (MM), and the percentage of water (W), considered to be result variables, were monitored and collected throughout the time by previously validated multifrequency bioelectrical impedance (BWB-800A, Tanita Corp. USA) [41]. This method is based on a 3-compartment model capable of evaluating BF, MM, and bone mineral content. Also, the percentage difference of each dependent variable was calculated throughout the weekly consultations, taking as a reference that recorded in the first one.

Likewise, the following independent variables were noted: age (years), height (cm), weight (Kg), and BMI (Kg/m2). The anthropometric measurements were taken following the recommendations in the standardized anthropometry handbook [42] by experienced personnel to diminish the coefficient of variation. Each measurement was taken three times, calculating the mean value. All the quantitative variables were measured with a precision of 0.1. For the height, a stadiometer (SECA 213) was used.

#### Physical activity

The strata proposed by Matthews [43] to evaluate the physical activity was employed. The MPA and IPA patients received instructions for doing aerobic exercises corresponding to a training-induced energy expenditure of approximately 300 to 600 kcal/day, while those relating to the LPA group did not receive any instructions in this respect. For the activity of MPA subjects, the women walked between 30 and 60 min daily or carry out a volume of steps of between 7500 and 10,000. To be considered as IPA individuals, the patients must undertake three times a week, intense physical activity sessions, above 70% of VO2max. Their heart rate (HR) was calculated using the Karvonen formula [44], and the maximum HR determined by the formula: 220 – age (years). Adherence was monitored by weekly exercise records completed by participants and researchers. In the MPA group, the controls were made by installing a pedometer (ACCUPEDO) via a mobile telephone application. The patient had to show her records weekly. The IPA group patients trained in the facilities of any sports center of their choice and can select among a variety of intense PA programs (CrossFit or Body Pump), which they visited three times a week, as well as completing the same steps objective of the MPAs.

#### Diet pattern

Concerning diet, the daily energy requirements was determined by estimating energy expenditure while at rest through the formula proposed by Harris-Benedict (655.0955 + 9.5634 [Weight (kg)] + 1.8496 [Height (cm)] – 4.6756 [Age (years)] [45] and multiplying the value obtained by a factor of 1.5 in those patients who were performing physical activities [46]. All the participants followed a dietary regime for 24 weeks with the following allocation of macronutrients: 25–30% proteins, 40–45% carbohydrates, and 30–35% fats. A hypocaloric diet was designed with a reduction of 500 kcal/day during the treatment period to obtain a weekly weight loss of 400 g. No vitamins or other nutritional complements are prescribed. After being included in the study, each woman took part in a 1-h seminar, in which a Dietitian-Nutritionist instructs them on the suitable selection and preparation of food. The menu proposed will be valid for seven days. The energy and nutritional supply were evaluated by the program Dietowin® and the weighing method [47].

The follow-up tests began the first week that the diet and physical activity were assigned. The body composition was measured after the night fast. Patients attempted to the clinic on the same day of the week, at the same time, and to wear the same clothes. The revision appointments continued at a weekly frequency up to week 24.

### Statistical analysis

The quantitative variables were presented with the mean and the standard deviation, whereas the qualitative ones in frequencies and percentages. To contrast, the goodness of fit to a normal distribution of data from quantitative variables, the Kolmogorov-Smirnov test with the Lilliefors correction was used. For the bivariant hypothesis contrast, the two-means Student-t test was made, while, for the qualitative variables, the Chi-test and Fisher’s exact test was performed when necessary. Likewise, for the three or more means analysis, the ANOVA repeated means test was used to evaluate the effects of the intervention at the baseline moment, at 3 and 6 months, and the correlation between the quantitative variables was verified by Pearson’s (r) correlation coefficient. The ANCOVA analysis of covariance was applied to determine the effect of the baseline data on the modification of body composition. Finally, in the case of not fulfilling the criterion of normality or homoscedasticity, the non-parametric versions of the tests mentioned were conducted. Adjusted linear regressions were made for each variable of the body composition (%BF and MM) and the weight at the final moment of the study to estimate the standardized Beta coefficients possessed by the PUSH notifications in the achievement of objectives. For all the statistical analyses, an alpha error probability of under 5% (*p* < 0.05) was accepted, and the confidence interval calculated with a 95% security. For the statistical analysis, the computer program IBM SPSS Statistics version 22.0 will be used.

## Discussion

The general objective of this protocol was to evaluate (1) the efficacy of PUSH notifications in an intervention aimed at improving the body composition of adult women who are overweight or obese, through dietary intervention, (2) analyzed the evolution of body composition based on PUSH notifications and prescribed physical activity. The intervention was evaluated through a randomized three-armed clinical test. It has been seen in the literature that the results of actions using mobile messaging via Push notifications could improve the degree of adherence to dietary prescriptions and physical activity, with different results. A significant number of women present physical activity levels below the minimum threshold recommend by official organizations. This sedentary lifestyle causes gains in total body weight and body fat. If the results of the assay demonstrate a positive effect, a new approach will be established based on the interaction of an APP and personal consultation, helping health professionals to establish real objectives in the prescription of physical activity and its follow-up in the patients who carry them out.

## Data Availability

Data sharing is not applicable to this article as no datasets were generated or analysed during the current protocol.

## Abbreviations

APP: Applications
BF: Body fat
BMI: Body mass index
IPA: Intense physical activity
LPA: Light physical activity
MM: Muscle mass
MPA: Moderate physical activity
PA: Physical activity
W: Water
